# Predictors of Irrational Parenthood Cognitions in an Iranian Group of Infertile Women

**DOI:** 10.1371/journal.pone.0070239

**Published:** 2015-03-10

**Authors:** Laya Farzadi, Aliyeh Ghasemzadeh, Zahra Bahrami-asl, Hossein Shirdel

**Affiliations:** 1 Women’s Reproductive Health Research Center, Tabriz University of medical sciences, Tabriz, Iran; 2 Alzahra University hospital, Tabriz University of medical sciences, Tabriz, Iran; 3 Department of Law, Tabriz Branch, Islamic Azad University, Tabriz, Iran; Azienda Policlinico S. Orsola-Malpighi, ITALY

## Abstract

**Objectives:**

The aim of this study was to investigate possible predictors of irrational parenthood cognitions among infertile women seeking treatment.

**Methods:**

In a cross-sectional study, 300 women who visited an Infertility Center in Iran during 2010 were studied. A pre-validated inventory was used to assess irrational parenthood cognitions. Potential predictors of the total irrational parenthood cognitions score were assessed.

**Results:**

Mean irrational parenthood cognition score was 39.7(Range 0–56). Through bivariate analysis, the score on irrational parenthood cognition was inversely correlated with age and positively correlated with length of time seeking for infertility treatment and length of time expecting pregnancy. In a multivariate model, infertile women with higher education, especially academic education, or those with higher economic status were less likely to have irrational parenthood cognitions. However, higher motherhood motivation, no previous experience of pregnancy and being under social pressure, from others around, increased the likelihood of having irrational parenthood cognitions.

**Conclusions:**

Some variables such as female spouse’s educational level and being under social pressure can independently predict irrational parenthood cognitions among infertile women that may be of use in designing health promotion plans in order to target the vulnerable women.

## Introduction

Approximately 10–15% of couples in their reproductive age involuntarily suffer childlessness. The reported figure has been reported as high as 33% in some studies[1,2]. It is known for a long time that problems arising from infertility are among the most devastating experiences of the affected couples[3]. Predicting various conditions in infertility through biological or non-biological factors has been a crucial matter of research in recent decades[4]. Mostly psychological aftermaths and affected quality of life are known to be of importance and women are more likely to be affected than are the men[5]. Childbearing is an important aspect of life for many couples and may even be the most important aspect for some eastern families. For most couples, the pregnancy and raising the children are among the most expected outcomes of their sexual relationship. Societal and parental pressures for propagation of the family name may be the source of a psychological burden for the infertile couple. Moreover, the physical, psychological, and financial challenges of assisted reproductive technology may have further impact on the couple[6]. Infertile women are even shown to be subject to high levels of physical, sexual and psychological violence [7]. Psychological assessment of infertile women has gained much attention and substantial amount of knowledge is available. Few studies have also addressed existence of irrational parenthood cognitions. In addition, a standard measurement scale for irrational parenthood cognitions is developed and validated in few languages[2,8–10]. Mostly, these studies have investigated effect of irrational cognitions on quality of life or development of psychological disorders and diseases. Nevertheless, the knowledge on predictors of irrational parenthood cognitions is quite scarce. The aim of this study was to investigate possible predictors of irrational parenthood cognitions among infertile women seeking infertility treatment.

## Materials/Patients and Methods

In this cross-sectional study, 300 women who visited the Infertility Center in Alzahra University hospital during 2010 were studied. This center is considered as a referral infertility center in North-West of Iran. Data were collected and made available using standardized questionnaires and the technical data were confirmed investigating medical files. Questionnaires were filled out for each of the participants in two parts. The first part included some background questions such as age, husband’s age, employment status, husband’s employment status, urbanity, educational level, husband’s educational level, duration of married life, and self-reported economic status. This part also included few questions regarding their infertility such as; duration of the waiting time for a pregnancy to occur, time seeking infertility treatment, their hope on treatment success, female infertility cause, having a previous pregnancy, previous live birth, social pressure by others around and the motherhood motivation.

The second part of the questionnaire belonged to the inventory developed to assess irrational parenthood cognitions among infertile women. Through this part, the participants were asked to score on a five point scale to what extent they agreed with these statements. The items were subsequently summated to a total score of 0–56. Higher scores indicated a stronger need to have children in order to live a happy life. The reliability of the English version of this scale, was earlier reported assessing internal consistency through Cronbach’s α, equal to 0.87 for female respondents[9]. The Persian version was also found to be reliable with Cronbach’s α above 0.7[10].

Data were entered into the computer and analyzed using STATA 11 statistical software package(STATA corporation, College Station, Texas 77845 USA). Primarily descriptive statistics were produced and bivariate analysis was done to explore possible correlates of irrational parenthood cognition score. Based on data distribution, Pearson or Kendall’s Tau correlation coefficients were calculated. To investigate the independent predictors of the cognition score, multivariate linear regression analysis was applied. Model diagnostics were checked. Due to existence of hetroscedastisity in distribution of error terms, regression analysis was rerun based on robust standard errors methodology. To make the regression coefficients comparable, standardized(beta) coefficients were also calculated. During the statistical analysis a p value<0.05 was considered as significant.

Study was approved by the committee of ethics in Tabriz University of medical sciences and written informed consent was obtained from all the participants.

## Results

Mean age of the participants was 28.4(SD 5.9) years. Mean age of the participant husbands was 33.6(SD 5.9) years. About 78% of the subjects were housekeepers and 17.4% were civil servants. Forty percent of the husbands were civil servants and 14.7% of them were workers. Women were married for a mean of 5.9 years(95% CI 5.3–6.5). The subjects were waiting to get pregnant for a mean of four years(95% CI 3.6–4.4). They were seeking for infertility treatment for a mean of 3.2 yeas(95% CI 2.7–3.6). Female infertility comprised 37.1% of the cases, male infertility was the cause in 15.4% and the remainder were either common cause or unknown. About 30% of the subjects reported a previous pregnancy, a quarter of which lead to a live birth and the remainder had ended in abortion or stillbirth.

About 40% of the participants said they are highly hopeful to get success in their treatment and about 13% said they have little hope or not hopeful at all. The economic burden of infertility treatment was described as high in 39.3%, moderate in 28.2%, and low or nothing in 31%. Five subjects didn’t give a clear answer to this question. Nearly 1/5^th^ of participants reported themselves as belonging to a good economic class and 19.3% classified themselves as low economic. The others considered themselves as middle economic class. One person didn’t answer this question.

**Fig 1:**
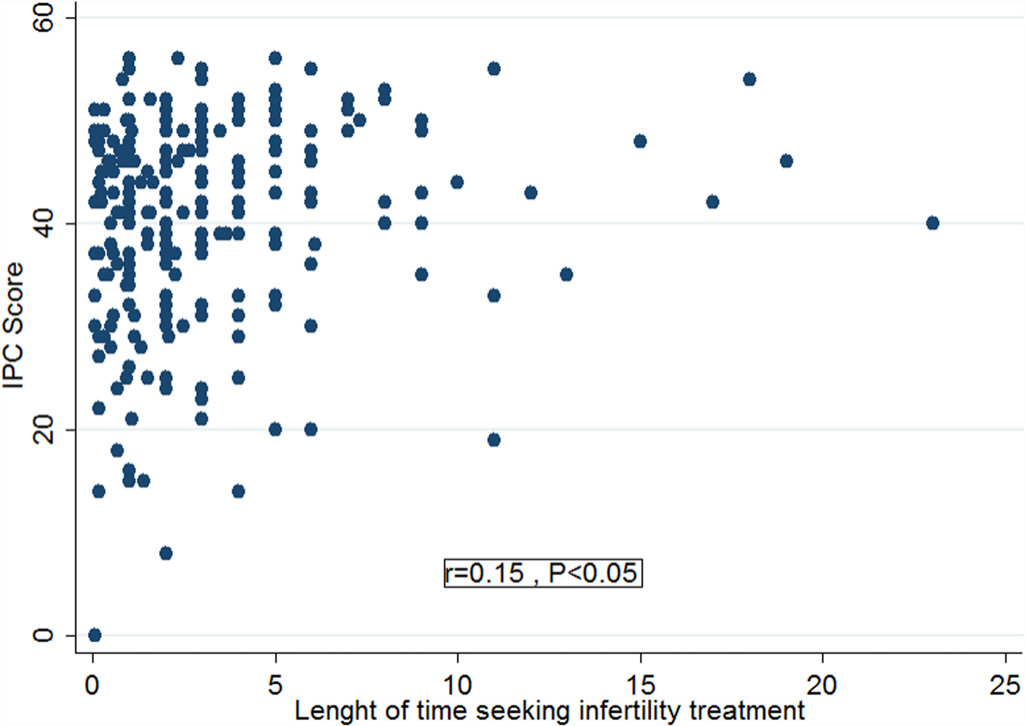
Scatter plot showing the correlation between the score of irrational parenthood cognitions (IPC) and the length of time seeking infertility treatment.

Mean irrational parenthood cognition score was 39.7(Range 0–56). The distribution of answers of participants to various items of the irrational parenthood cognitions scale is given in Table 1. Using Pearson correlation assessment, the score on irrational parenthood cognition was inversely correlated with age and positively correlated with duration of the time seeking for infertility treatment and length of time expecting pregnancy. The observed linear correlations coefficients were found to be weak but statistically significant (P<0.05). However, as can be found in Fig. 1 such correlations may not be linear. Using Kendall’s Tau correlation assessment, the score on irrational parenthood cognition was weakly correlated with variables such as: how intensely was the economic status of couples affected by infertility costs, how big was their demand to get pregnant, how large was the social pressure imposed by relatives and people around, how low was the economic level, and how less was their hope to success(P<0.05). The score on irrational parenthood cognition was weakly correlated with level of education both for the women and their husbands.

**Table 1:** Items of irrational parenthood cognition questionnaire and the answers of participants to them.

Item	Totally agree	Agree to some extent	No idea	Disagree to some extent	Totally disagree
Having a child is the most important thing in life	167(55.7)	109(36.3)	15(5)	1(0.3)	8(2.7)
A life without children is useless and empty	109(36.3)	101(33.7)	40(13.3)	23(7.7)	27(9)
It is absurd that some people can have children quite easily, while others never do	84(28.1)	56(18.7)	32(10.7)	61(20.4)	66(22.1)
The whole world revolves around children	95(31.8)	86(28.8)	38(12.7)	40(13.4)	40(13.4)
Friends have no idea what people go through who cannot have children	180(60)	62(20.7)	27(9)	21(7)	10(3.3)
It is impossible to understand that some women decide to have an abortion	107(36.1)	56(18.9)	46(15.5)	49(16.5)	39(13.1)
You start feeling inferior when you cannot have children	96(32.1)	85(28.4)	25(8.4)	35(11.7)	58(19.4)
You start hating your body when you cannot have children	67(22.4)	70(23.4)	32(10.7)	35(11.7)	95(31.8)
An IVF treatment is extremely heavy and painful	135(45)	108(36)	22(7.3)	22(7.3)	13(4.3)
Your whole world is destroyed when you/your partner have/has your/her period after replacement of the embryos	181(60)	69(23.1)	19(6.4)	18(6)	12(4)
The waiting during an IVF cycle puts you through hell	144(48.2)	91(30.4)	22(7.4)	24(8)	18(6)
One’s whole world is destroyed when the last IVF treatment fails	190(63.3)	53(17.7)	24(8)	16(5.3)	17(5.7)
Not having children causes lifelong suffering	148(49.5)	94(31.4)	26(8.7)	18(6)	13(4.4)
One would want to do anything to get pregnant	152(50.8)	70(23.4)	15(5)	21(7)	41(13.7)

Row percentages are given inside parentheses

Independent predictors of irrational parenthood cognitions score were explored using a multivariate linear regression model. The model coefficients are given in Table 2. Other correlates of irrational parenthood cognition score in bivariate analysis were excluded from the final regression model. The model was found to predict 31% of variability in irrational parenthood cognition score.

**Table 2:** Multivariate regression results predicting irrational parenthood cognition score based on robust standard errors.

Predictors	Coefficients	t	P value	Standardized coefficients
Primary school education(wife’s)	Reference	-	-	-
Guidance school education(wife’s)	-1.3	-0.8	0.4	-0.05
High school education(wife’s)	-3.7	-2.9	0.005	-0.18
Academic education(wife’s)	-9.6	-6.2	0.0001	-0.43
No previous pregnancy	4	3.3	0.001	0.18
Lower economic status(couple’s)	1.6	1.9	0.053	0.1
Lower motivation for pregnancy(wife’s)	-2.6	-3.5	0.001	-0.193
Lower social pressure from others around	-1.6	-3.5	0.0001	-0.187
Constant	42.3	11.6	0.0001	-

## Discussion

A strong association between irrational parenthood cognitions and depression has been reported in literature[11–13].Moreover, it is stated to be a major predictor of quality of life among infertile women[2,8,9,14,15]. Contrary to most of the earlier studies, the present study was specifically focused on assessing predictors of irrational parenthood cognitions rather than its aftermaths as was addressed in previous research.

In a multivariate model, the authors found that infertile women with higher education, especially academic education, or those with higher economic status were less likely to have irrational parenthood cognitions. However, higher motherhood motivation, no previous experience of pregnancy and being under social pressure from others around increased the likelihood of having irrational parenthood cognitions. The effect of education, especially academic education, can mainly be explained at least by two factors as, having higher overall insight, and also greater life independence that may affect their mental reactions. Higher education and higher economic level can also be indicators of living at a higher social class level. Regarding the finding on previous experience of pregnancy, and social pressure, maybe stress and seeking coping strategies play a role. However due to paucity of knowledge in this field further research is necessary to confirm the role of these predictors and clarify plausibility of associations.

As can be found in results of this study, in bivariate analysis, the score on irrational parenthood cognition was inversely correlated with age and positively correlated with length of time seeking for infertility treatment and length of time expecting pregnancy. Fekkes et al. also found that younger women had higher levels of irrational parenthood cognitions than older women. However, in our study this association was weak and disappeared in multivariate analysis. This can be due to lower variability of the age or confounding effect of other variables that were found to be independent predictors of irrational cognition score and controlled in multivariate analysis. For example older people are more likely to have a previous pregnancy which in turn was found to be inversely associated with irrational parenthood cognition. Another theoretical explanation can be that in some cultures infertility leads to divorce or polygamy decreasing the likelihood of being captured at older ages during the sampling in clinic based studies. Nevertheless, this may not be much applicable in this context. Polygamous marriages are not popular and are even criticized in Iran; but in some Arabic and African countries, they are common and are related to the psychological outcomes of infertility and infertility treatment-seeking behaviors[8,14,16]. Divorce due to infertility is also not reported to happen before seeking for infertility treatment for a socially acceptable length of time.

One major question could be that why a problematic cognitive status is important. The answer lies in its association with quality of life and, psychological disorders, as stated before, and also possible negative health care seeking behaviors affecting the treatment process. Interestingly of importance can also be the consequent change in immune response that is reported to occur due to psychological aftermaths of infertility [10,11,14,16–18].

Therapeutic counseling is recommended to be initiated before infertility treatment in order to improve coping skills of the patients[9]. Cognitive therapy has been shown to be a reliable alternative to pharmacotherapy and superior to fluoxetine in the resolution or reducing of depression and anxiety of infertile women[19]. Its role may be even larger when used for women who have a poor score regarding irrational parenthood cognitions. Future research is recommended to consider comparing efficacy of cognitive therapy between those suffering psychological aftermaths of infertility with higher-versus-lower scores of irrational parenthood cognition.

Screening infertile women regarding irrational parenthood cognitions and assessing their psychological status may prove beneficial to improve quality of life among infertile women. Specific populations screened through predictors of irrational parenthood cognition can be targeted in case of resource limitations in implementing psychological support teams.

## Conclusion

Some variables exist that can independently predict irrational parenthood cognitions among infertile women that may be of use in designing health promotion plans targeting vulnerable women.
